# Mathematical Model to Assess Mutational Burden in Retinal Dystrophy Patients Negative to Mendelian Genetic Tests and Carriers of Multiple Recessive Variants

**DOI:** 10.18502/jovr.v21.18236

**Published:** 2026-08-26

**Authors:** Maria Chiara Medori, Alessandro Macchia, Paolo Enrico Maltese, Jan Miertuš, Francesca Cristofoli, Ornela Gordani, Xhilda Dhamo, Eglantina Kalluci, Dominika Vešelényiová, Stanislav Miertuš, Iveta Dirgová Luptáková, Jiří Pospíchal, Rashid Ahmed, Andrea Bernini, Giorgio Placidi, Benedetto Falsini, Pietro Chiurazzi, Dror Rosentraub, Matteo Bertelli

**Affiliations:** ^1^MAGI'S LAB, Rovereto, Italy; ^2^Department of Biotechnology, Chemistry and Pharmacy, University of Siena, Siena, Italy; ^3^MAGI EUREGIO, Bolzano, Italy; ^4^Department of Applied Mathematics, Faculty of Natural Sciences, University of Tirana, Albania; ^5^Department of Biology, Institute of Biology and Biotechnology, Faculty of Natural Sciences, University of Ss. Cyril and Methodius, Trnava, Slovakia; ^6^Department of Biotechnology, Institute of Biology and Biotechnology, Faculty of Natural Sciences, University of Ss. Cyril and Methodius in Trnava, Trnava, Slovakia; ^7^International Centre for Applied Research and Sustainable Technology, Bratislava, Slovakia; ^8^Institute of Computer Technologies and Informatics, Faculty of Natural Sciences, University of Ss. Cyril and Methodius, Trnava, Slovakia; ^9^Nick Holonyak Jr. Micro and Nanotechnology Laboratory, University of Illinois, IL, USA; ^10^Ophthalmology Unit, Fondazione Policlinico A. Gemelli, Rome, Italy; ^11^Università Cattolica Sacro Cuore, Rome, Italy; ^12^Medical Genetics, Fondazione Policlinico Universitario Agostino Gemelli IRCCS, Rome, Italy; ^13^Genomic Medicine, Università Cattolica del Sacro Cuore, Rome, Italy; ^14^MAGISNAT, Atlanta Tech Park, Peachtree Corners, GA, USA; ^15^MCM and AM contributed equally to this work.

**Keywords:** Retinal Dystrophies, Mutational Burden, Digenic Inheritance, Protein–Protein Interaction Networks, Retinoid Cycle Pathway

## Abstract

**Purpose:**

Despite advancements in genetic testing, a significant number of patients with retinal dystrophy receive negative test results due to single-allele mutations in genes with autosomal recessive inheritance patterns. This study aimed to address this diagnostic challenge by proposing a preliminary mathematical model based on mutational burden analysis. This approach can identify unique digenic or oligogenic combinations, potentially prioritizing them for further investigation.

**Methods:**

We retrospectively analyzed clinical and genetic data from 527 subjects comprising 453 patients with retinal dystrophies (cases) and 74 controls and created semi-synthetic datasets. Protein–protein interaction networks were analyzed, and the significance of mutational burden in specific molecular pathways was investigated.

**Results:**

Our model identified two metabolic pathways that are highly correlated to each other, namely “retinoid cycle disease events” and “canonical retinoid cycle in rods (twilight vision).” These pathways were significantly impacted by the burden of variants we found in the case group. Moreover, the gene combinations (*ABCA4, RDH12*) and (*ABCA4, RBP3*) were identified exclusively in cases of retinal dystrophies.

**Conclusion:**

The proposed preliminary model provides a systematic approach to calculate the mutational burden in patients with retinal dystrophies and previous negative genetic test results. Moreover, the bioinformatic analysis facilitated the identification of recurrent combinations of involved genes, suggesting possible new digenic or oligogenic inheritance patterns.

##  INTRODUCTION

Retinal dystrophies are a group of inherited eye disorders characterized by progressive degeneration of the retina, leading to vision loss. These conditions can result from genetic mutations affecting multiple genes and encompass a wide range of inheritance patterns, including autosomal dominant, autosomal recessive, and *X*-linked conditions, each caused by mutations in different genes that ultimately disrupt the function of retinal cells and produce a similar clinical outcome.^[1,2,3,4]^ The complex nature of retinal dystrophies stems from many elements, including locus heterogeneity. Proteins encoded by genes featuring locus heterogeneity exhibit high interconnectivity within the human protein interaction network and tend to form clusters.^[5]^ Up to 30% of locus heterogeneity cases involve disease-causing proteins that are members of the same protein complex.^[6]^ This clustering suggests that these genes participate in similar biological processes and pathways.^[5]^ Retinitis pigmentosa, a retinal dystrophy with over 70 known causative genes, exemplifies this phenomenon.^[7]^ Network analysis of retinitis pigmentosa genes and their interacting partners has revealed potential new disease candidates and provided insights into the etiopathology of the disorder.^[7]^ Understanding these protein–protein interactions and molecular pathways is crucial for elucidating the mechanisms underlying locus heterogeneity.^[7,8]^

Traditional genetic testing based on Mendelian inheritance in hereditary retinal dystrophies (IRDs) has limitations in accurately diagnosing the diverse forms of IRDs. Recently, it has become evident that additional modes of inheritance, beyond the classic Mendelian framework, may contribute to retinal diseases. Indeed, retinal dystrophies can also result from the interaction of single-allele variants across multiple genes, which are then manifested as digenic, trigenic, and oligogenic inheritance patterns.^[9,10,11,12]^ These concepts bridge the gap between Mendelian and multifactorial traits, offering insights into how multiple genetic variations can converge to influence phenotypic outcomes. Oligogenic inheritance can affect not only retinal dystrophies but also related syndromes such as Usher syndrome (type ID), Usher syndrome (type IIC), Joubert syndrome 9, Joubert syndrome 15, Methylmalonic aciduria and homocystinuria, cblC type, and Bardet-Biedl syndrome [Table 1]^[13,14,15,16,17,18,19,20]^

**Table 1:** Digenic and oligogenic diseases affecting the retina and vision

**Disease**	**OMIM**	**Gene**	**Inheritance pattern**	**Reference**
Retinitis pigmentosa 7	608133	ROM1-RDS	Digenic	13
Retinitis pigmentosa	-	RP1L1-C2orf71	Digenic	14
Usher syndrome, type ID	601067	PCDH15-CDH23	Digenic	15
Usher syndrome, type IIC	605472	ADGRV1-PDZD7	Digenic	16
Joubert syndrome 9	612285	CC2D2A-CEP41	Digenic	17
Joubert syndrome 15	614464	CC2D2A-CEP41	Digenic	18
Methylmalonic aciduria and homocystinuria, cblC type	277400	MMACHC-PRDX1	Digenic	19
Barde-Biedl Syndrome	–	BBS1-BBS2-BBS4-BBS5-BBS6-BBS7-BBS9-APOE-C8orf37-CEP290-DNAAF1-ESR1-GHR-GUCY2C-MC4R-MCHR1-MKS1-NCOA2-NPC1-PDX1-RYR1-STRA6-ZNF423	Oligogenic	19
Familial exudative vitreoretinopathy	–	FZD4-LRP5	Digenic	20
This table presents examples of digenic and oligogenic diseases that impact retinal function and vision. It includes the disease name, OMIM number, associated genes, mode of inheritance, and relevant references.				

Oligogenic diseases have been studied using a mutational burden model to detect their possible genetic causes.^[21]^ The Digenic Diseases Database (DIDA) contains information on 44 digenic diseases, comprising 213 digenic combinations.^[22]^ In a study of 124 digenic combinations from DIDA, 31% were true digenic instances, meaning both genes were required for developing the disease. The remaining 69% were composite cases, where a variant in one gene was sufficient to produce the phenotype, but an additional variant in a second gene impacted the disease phenotype or altered the age of onset, a phenomenon called epistasis.^[22]^ Another study used a mutational burden model to identify digenic interactions in human diseases and developed the Digenic Interaction Effect Predictor (DIEP). DIEP is a machine-learning approach that achieves high accuracy and sensitivity in independent testing datasets, outperforming other gene-based digenic interaction predictors.^[23]^ Finally, mutational burden has been proposed as a factor involved in many genetic diseases, suggesting that the combined impact of rare genetic variants plays a role in modulating disease phenotype and contributing to variability in clinical expression.^[24,25]^

The complexity of retinal dystrophies—stemming from the complex genetic landscape, variable symptoms, and diverse inheritance patterns—underscores the need for new diagnostic methods. Patients with genetic disorders can exhibit multiple variants in single alleles of different recessive genes, leading to the production of altered proteins that may contribute to the manifestation of the disorder.^[26]^ The proteins produced by these mutated genes often interact within the same biochemical pathway, adding complexity to the diagnostic process.^[27,28,29]^ The presence of multiple variants in a single pathway can create a complex interplay of genetic and environmental factors that influence the severity and presentation of the disorder.^[30]^

Advanced computational approaches, such as machine learning, are becoming increasingly crucial tools for analyzing complex genetic data and predicting disease outcomes, and they could be used to analyze the complex interplay among genes, gene–protein interactions, and protein–protein interactions.^[23]^ When the Mendelian inheritance model is applied, a substantial proportion of cases (25– 40%) yield negative diagnostic results.^[31,32]^ We hypothesize that a subset of the negative results for the Mendelian inheritance model may test positive when other models of inheritance are considered.

This study aims to evaluate whether in Mendelian negative subjects there may be a mutational accumulation significantly associated with the observed pathological phenotype. Moreover, this study seeks to identify unique genetic combinations that may be proposed as new oligogenic inheritance patterns for future studies. Specifically, we investigated which pathways are enriched among the genes in which we identified heterozygous and recessive variants. Subsequently, we analyzed which of these pathways were more prevalent in cases than in controls. To investigate these hypotheses, we developed a mathematical model to calculate mutational burden and select genetic combinations that can be interpreted with oligogenic inheritance patterns.

##  METHODS

### Data Collection

A dataset was constructed retrospectively using demographic data, genetic data, and specific ophthalmologic data. For clinical diagnosis in Italy, patients are recruited by clinical specialists for heredodegenerative diseases in several hospitals and then undergo genetic analysis in MAGI laboratories. The dataset of this study includes patients who were clinically diagnosed with IRDs, but the results of their Mendelian genetic testing were negative. These patients were referred to as cases. Another dataset of patients, referred to as controls, included individuals diagnosed with other genetic diseases but with a genetic panel comprising IRD-related genes as well.

The datasets included anonymously coded information such as age, genetic variants, nucleotide, amino acid, “ACMG classifications,” visual field (VF) in the right eye, VF in the left eye, and mean VF. Genetic variants have been classified according to the ACMG guidelines as pathogenic, likely pathogenic, and variant unknown significance (VUS HOT).^[33]^ The patients were informed about the significance of genetic testing, and we obtained written informed consent from all of them in accordance with the Declaration of Helsinki. Ethical approval and clearance were received from the Ethical Committee of Azienda Sanitaria dell'Alto Adige, Italy (Approval No. 132-2020), which covers the retrospective use of genetic and clinical data, for research purposes, obtained during diagnostic practice.

### Data Processing and Data Transformation

For each ACMG interpretation, we assigned a value based on the effect of the variant on the phenotype. Therefore, we assumed a variant classified as “Pathogenic” or “Likely Pathogenic” to have a higher role in the possible occurrence of the disease than a “VUS.” ACMG classifications were assigned arbitrary weights based on their pathogenicity levels (*P* = 0.5, LP = 0.3, VUS HOT = 0.2) and normalized by age to account for potential age-related biases.

The data processing pipeline was designed to normalize the “pathogenicity weight” assigned to each variant by patient age, map mutated genes to metabolic pathways, and perform statistical comparisons between the two groups. (The overall workflow is summarized in Figure 1.) Normalized pathogenicity weights were integrated into the dataset, which included patient ID, mutated gene, ACMG classification, mean VF evaluation (VF mean), and date of birth. This normalization ensured that the impact of age on genetic evaluation was minimized before further analysis. Clinical data regarding VF were missing for the control patients. Since these individuals were not diagnosed with any retinal diseases, we assumed their VF to be 10. In the case group, missing values of VF were substituted with random values ranging from 1 to 7. Considering these final steps, the datasets are referred to as semi-synthetic throughout the article.

A dictionary was constructed to map genes to their corresponding metabolic pathways using publicly available databases, including KEGG and Reactome, as well as STRING, an open-source tool used to retrieve enriched pathway information for genes with at least one variant in the dataset [Table S1]. Mutated genes identified in the case group were systematically mapped to these metabolic pathways to enable detailed pathway enrichment analysis. Dummy columns were added to the main data frame to represent the mutated genes and their associated pathways, facilitating downstream calculations.

Once pathogenicity weights were normalized, they were multiplied by the corresponding values in the metabolic pathway columns to calculate a weighted score that represents the contribution of each gene to its associated pathways. The data were then grouped by patient ID, with the pathway scores and pathogenicity weights aggregated by summing the metabolic pathway data and averaging the pathogenicity weights. This step provided a consolidated view of each patient's genetic and pathway-level data. Mean values for each metabolic pathway were also calculated separately for the case group (patients with retinal dystrophies) and the control group.

Finally, a Mann-Whitney U test was performed to compare metabolic pathway scores between the case and control groups. This nonparametric test was chosen to account for the non-normal distribution of the data, enabling robust statistical comparisons of the mean values for each pathway. The results of this analysis highlighted pathways with significant differences between the two groups, shedding light on the potential role of metabolic pathways in the disease pathogenesis.

**Figure 1:**
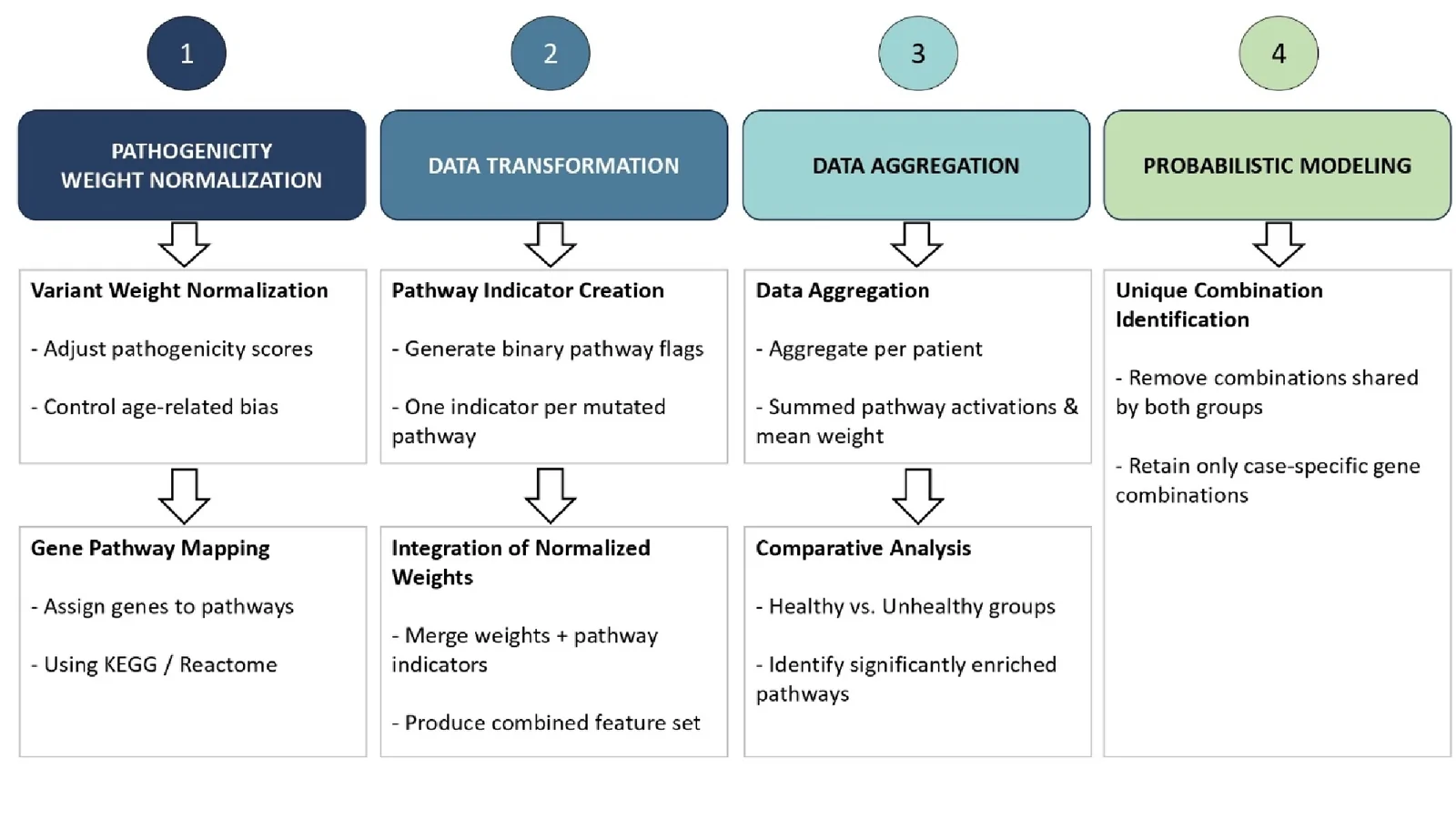
Flowchart of data processing and mathematical model development illustrating the steps involved in developing the mathematical model, including “Pathogenicity weight” normalization, data transformation, data aggregation, and probabilistic modeling.

### Statistical Analysis

Statistical analyses were performed using the Mann-Whitney U test and the Shapiro-Wilk test to evaluate data distributions, correlations, and significance levels. The Mann-Whitney U test was applied to assess differences between gender and key variables, including MEAN LE+RE (LE: left eye; RE: right eye), mutated gene counts, and age. Correlation analysis was conducted to examine relationships between variables, with heatmaps constructed to visualize interactions within the dataset. Python libraries like Pandas, NumPy, Seaborn, and scikit-learn were used for statistical analysis and data visualization. The results were considered statistically significant when the *P*-value was less than 0.05.

### Probabilistic Kernel Density Estimation

Kernel density estimation (KDE) was used to quantify the association between mutational burden and retinal conditions, specifically focusing on individual pathways. To ensure comparability across the cohorts, feature values were standardized to have a mean of zero and a standard deviation of one. To determine the optimal smoothness of the KDE model, a rigorous cross-validated grid search was conducted to pinpoint the optimal bandwidth parameter. With the optimal bandwidth identified, the KDE model was fitted to the standardized feature values, and a comprehensive evaluation of the density was performed across a range of values. A key component of the analysis involved the calculation of percentile ranks for each patient's standardized feature value. A threshold was established at the 90 ${}^{\text{th}}$ percentile in order to classify patients based on their mutational burden. Specifically, mutational burden is associated with the retinal condition in patients exhibiting higher feature values surpassing the threshold.

##  RESULTS

### Demographic and Clinical Characteristics

The demographic characteristics of the cohort, consisting of 527 patients divided into cases and controls, are reported in Table 2. The case group included 453 individuals, while the control group consisted of 74 individuals. Among the cases, 53% were males and 47% were females, compared with the control group, which was composed of 69% males and 31% females. The age range of cases spanned from 11 to 102 years, with a mean age of 47 ± 19 years. The controls had an age range from 8 to 86 years, with a mean age of 46 ± 20 years. Cases showed a mean LE+RE of 4.44 ± 1.38, whereas controls exhibited a significantly higher score of 10 ± 0. This is in line with the construction criteria of the semi-synthetic dataset. The pathogenicity weight score was higher in cases (0.38 ± 0.13) compared to controls (0.18 ± 0.03). It indicates that variants in the controls were, on average, less pathogenic, although patients in this group had a higher average number of mutated genes compared to the cases (4.96 ± 1.77 vs 2.52 ± 1.67).

**Table 2:** Demographic and clinical characteristics of the analyzed cohort

**Characteristic**	**Cases (** ***n*** ** = 453)**	**Controls (** ***n*** ** = 74)**	***P-*** **value**
Gender (Male/Female)	238/215 (53%/47%)	51/23 (69%/31%)	< 0.05
Mean Age (years, Min–Max ± SD)	47 (11 – 102) ± 19	46 (8 – 86) ± 20	> 0.05
Mean LE+RE ( ± SD)	4.44 ± 1.38	10 ± 0	< 0.05
Mutated gene count	2.52 ± 1.67	4.96 ± 1.77	< 0.05
Pathogenicity weight score	0.38 ± 0.13	0.18 ± 0.03	< 0.05
This table summarizes the demographic information and clinical data of the cohort analyzed in the study.			

The Mann-Whitney U test indicated no statistically significant difference between age distributions in the two groups (*P* = 0.617). The similar age distributions between the control and case groups suggest that age is not a confounding factor in comparison to other variables such as pathway activations and pathogenicity weight scores.

The chi-square test of independence indicated a statistically significant difference in the gender distribution between the control and case groups. The higher proportion of males in the case group suggests a potential gender-related factor in the prevalence of retinal conditions, warranting further investigation.^[34,35]^

### Correlation Analysis between Retinal Pathways and Visual Loss

Correlation analysis was performed to assess the relationship between different molecular pathways. Our analysis revealed significant correlations (positive correlations with *r *values near 1.0) between several key genetic pathways associated with retinal conditions [Table 3].

### Mann-Whitney U Test to Determine Pathways with Higher Values in the Case Group

The scatter plot in Figure 2 illustrates the* P*-values of various pathways, comparing their activation levels between control and case groups. Pathways with lower *P*-values indicate greater differences in activation between the groups. The *P*-values are plotted on a logarithmic scale to highlight the most significant pathways. Key findings include significant differences in the “canonical retinoid cycle in rods (twilight vision)” (*P* = 0.012084) and “Retinoid cycle disease events” (*P* = 0.00002), marked in red. Other noteworthy pathways, such as visual phototransduction, sensory perception, and retina homeostasis, also showed significant differences and are marked in blue.

### Probabilistic KDE to Quantify the Association of Mutational Burden with Retinal Conditions

We used KDE to quantify the association between mutational burden and retinal conditions based on significant genetic features. This was achieved with a focus on the "canonical retinoid cycle in rods (twilight vision)," despite its high correction with the “retinoid cycle disease events” pathway [Table 3]. The KDE method was used to estimate the probability density distribution of significant genetic features linked to retinal conditions. The establishment of a 90 ${}^{\text{th}}$ percentile threshold facilitated the classification of patients. This is visually represented in Figures 3A and 3B, where the KDE curve delineates the density distribution of datapoints, and a vertical dashed line indicates the set threshold. Areas of higher density highlighted by the KDE curve signify a larger concentration of patients with similar feature values, thereby enhancing the interpretability of our results.

### Identification of Unique Gene Combinations in the Case Group

Apart from proposing a method for calculating mutational burden, our model enabled us to identify which gene combinations were present in cases compared with control patients. Considering that the “retinoid cycle disease events” and “the canonical retinoid cycle in rods (twilight vision)” were the two most statistically significant pathways, that they are highly correlated, and that the genes in the former all exist in the latter, we decided to study only the combinations present in the twilight vision pathway. Firstly, we selected all patients who had at least one variant in at least one gene within the twilight vision pathway, resulting in 207 cases and 27 controls. Secondly, we calculated all the possible genetic combinations identified in the analyzed cohorts. Finally, we considered only the genetic combinations present exclusively in the case group (see the model flowchart in Figure 4). Our analysis indicated that the combinations (*ABCA4, RDH12*) and (*ABCA4, RBP3*) were specific to the cases. Besides, the incidence of combinations in cases compared to controls was not statistically different according to Fisher's exact test [Table 4].

**Figure 2:**
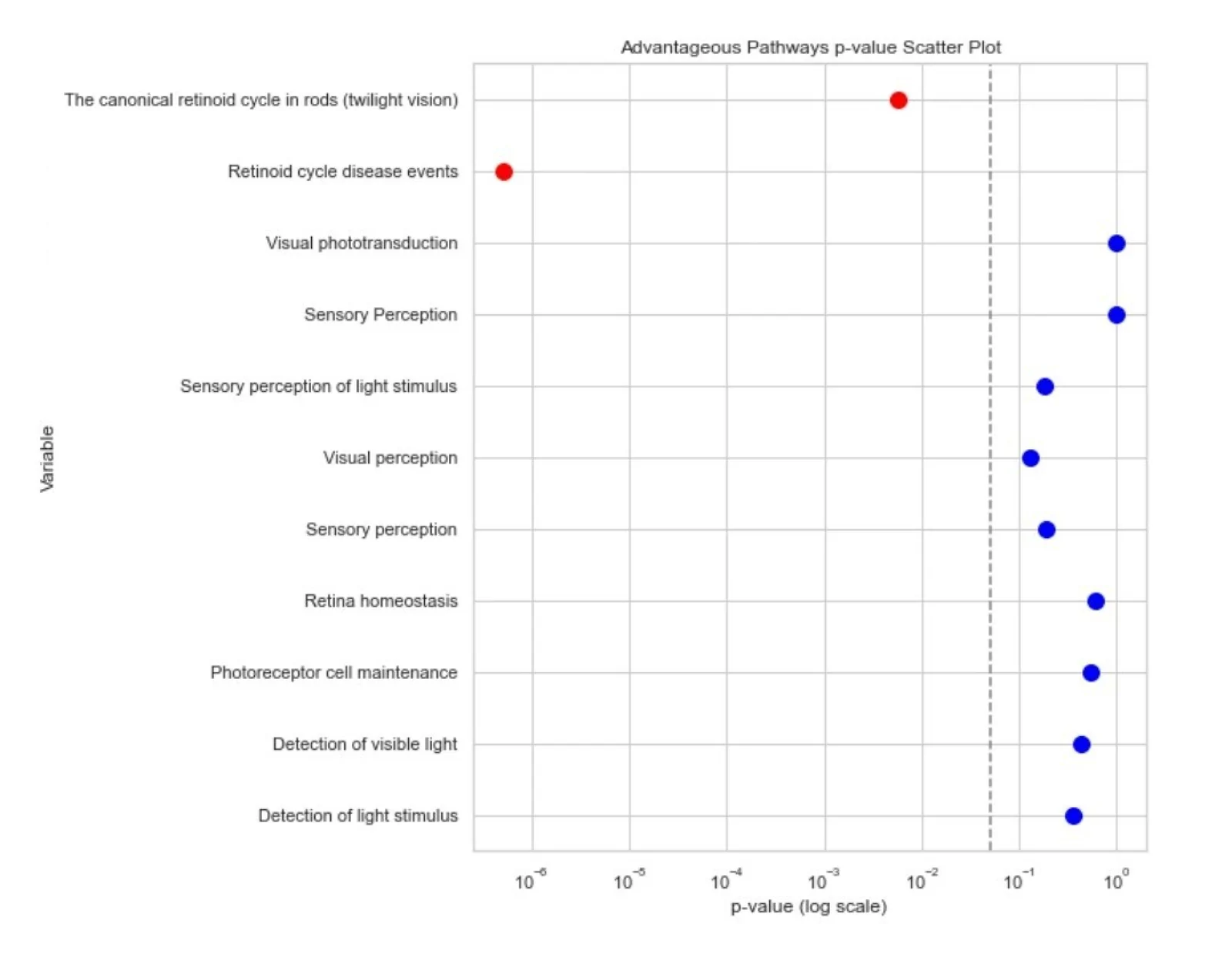
Unhealthy group advantageous pathways. This figure shows the result of the Mann-Whitney U test to compare the data from the interest group and the control group for each of the identified advantageous metabolic pathways. The two pathways that result significantly are: the canonical retinoid cycle in rods (twilight vision): *P* = 0.012084. Retinoid cycle disease events: *P* = 0.000002. The *P*-values are plotted on a logarithmic scale to highlight the most significant pathways.

**Table 3:** Correlation analysis between retinal pathways and the proposed scientific rationale for the correlations

**Pathway**	***r value***	**Possible scientific rationale**
Retinoid Cycle Disease Events and the Canonical Retinoid Cycle in Rods (Twilight Vision)	0.92	Abnormalities in the retinoid cycle are closely associated with the development and progression of retinal diseases.
Visual Phototransduction and Sensory Perception of Light Stimulus	0.99	Modulating phototransduction could influence sensory responses.
Visual Perception and Sensory Perception	0.99	Improvements in visual perception could positively impact broader sensory capabilities.
Photoreceptor Cell Maintenance and Retina Homeostasis	0.74	The maintenance of photoreceptor cells is essential for the overall health and homeostasis of the retina.
Summary of the correlation analysis between different retinal pathways, highlighting statistically significant associations and their potential scientific rationale. The table provides insights into how these pathways may interact and contribute to retinal function or disease mechanisms.		

**Figure 3:**
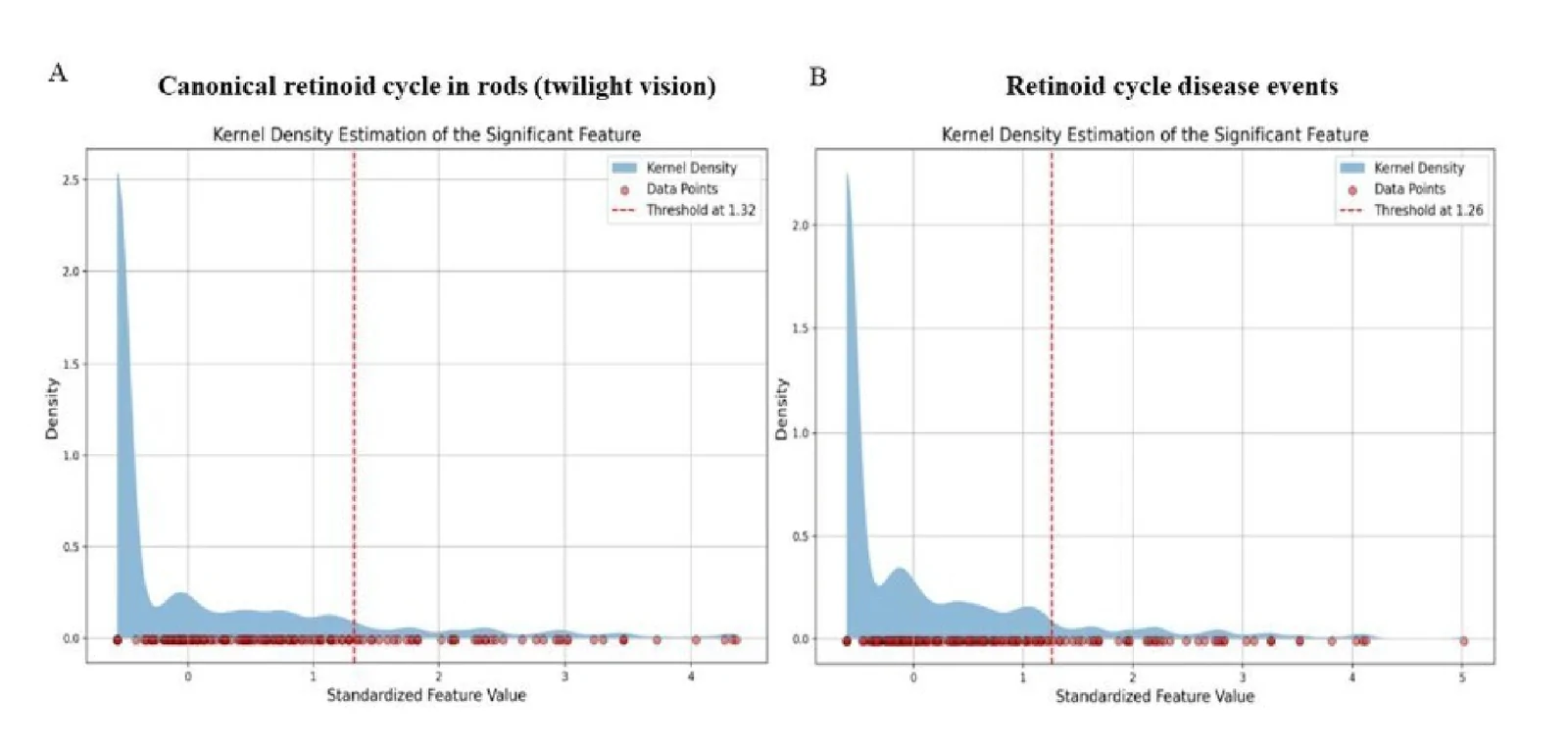
Kernel Density Estimation (KDE) of retinoid cycle disease events. The KDE curve delineates the density distribution of datapoints, and a vertical dashed line indicates the set threshold for patient classification. The *X*-axis represents the percentile rank estimated by the KDE for the average of the “pathogenicity weight” that is summed for each variant in the target pathway, while the *Y*-axis indicates the estimated density. For higher accuracy in the significant pathway result, we use both Kernel density and the Mann-Whitney U test. (A) The KDE based on the canonical retinoid cycle in rods (twilight vision) pathway. (B) The retinoid cycle disease events pathway.

**Figure 4:**
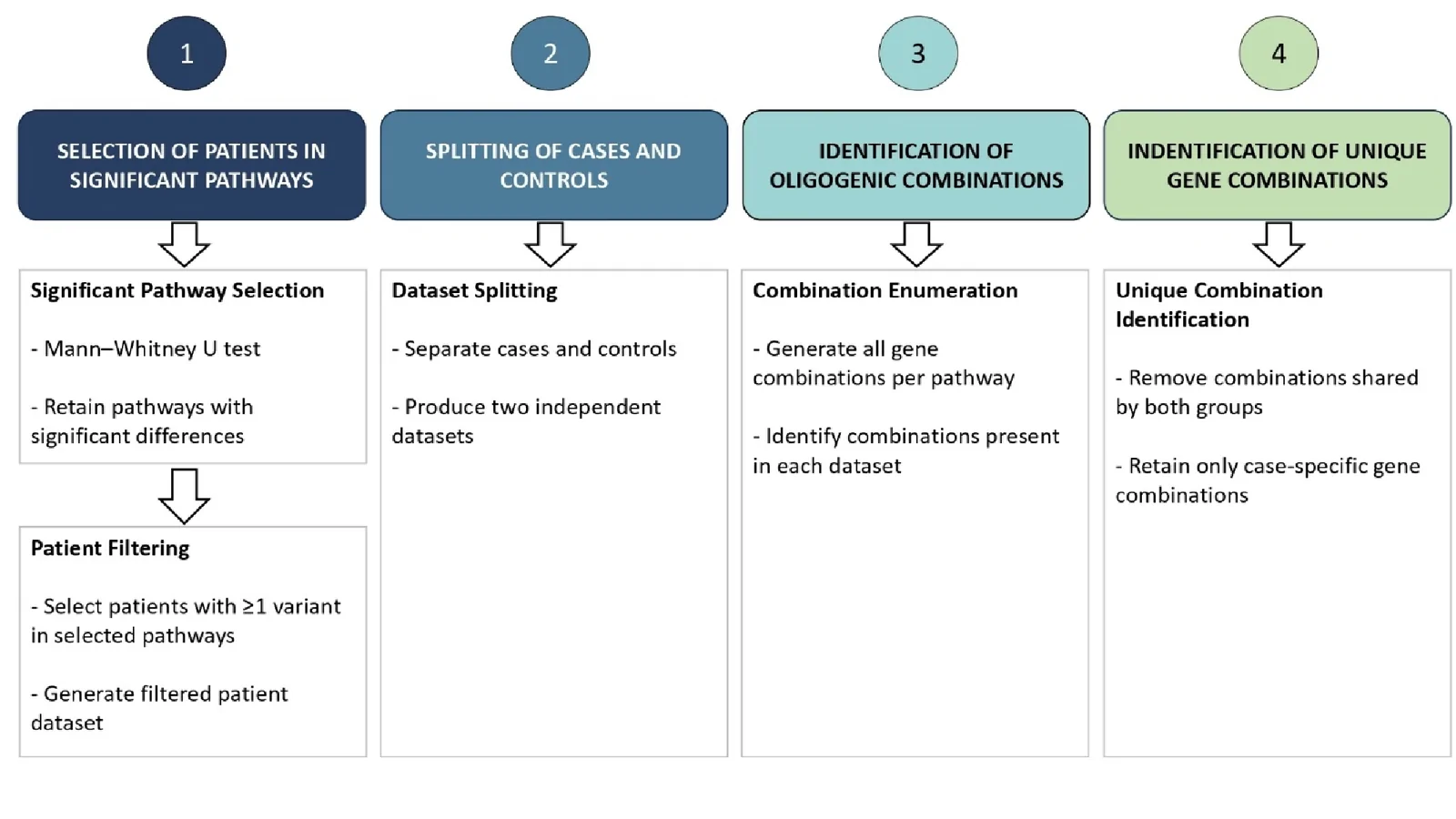
Flowchart of the model for the identification of unique gene combinations present in unhealthy patients but absent in controls. The process includes selecting patients with variants in genes from the twilight vision pathway, calculating genetic combinations in the analyzed cohorts, and selecting combinations exclusive to unhealthy patients.

**Table 4:** Gene combinations in the twilight vision pathway present exclusively in the case group

**Combination**	**Gene 1**	**Variant 1**	**Gene 2**	**Variant 2**	**No. of patients**
*ABCA4, RBP3*	*ABCA4*	NM_000350.3:c.5603 A > T	RBP3	NM_002900.3:c.3715C > T	1
*ABCA4, RBP3*	*ABCA4*	NM_000350.3:c.6148G > C	RBP3	NM_002900.3:c.3715C > T	2
*ABCA4, RDH12*	*ABCA4*	NM_000350.3:c.766G > T	RDH12	NM_152443.3:c.C184C > T, NM_152443.3:c.805_809del	3
*ABCA4, RDH12*	*ABCA4*	NM_000350.3:c.3936G > C, NM_000350.3:c.574G > A	RDH12	NM_152443.3:c.578G > A	4
List of gene combinations within the twilight vision pathway found exclusively in cases and absent in controls. This table highlights potential oligogenic combinations associated with retinal dystrophies and provides insights into the molecular mechanisms underlying retinal dysfunction.					

##  DISCUSSION

Retinal dystrophies comprise a heterogeneous group of genetic disorders that are characterized by progressive degeneration of the retina, resulting in vision loss and impairment.^[1]^ Conventional genetic testing methods, such as targeted sequencing panels and whole-exome sequencing, have significantly expanded our knowledge of retinal dystrophy-associated genes, but many genetic tests based on the Mendelian model of inheritance still result as negative.^[5,6]^

Considering that the mutational burden has been proposed to be correlated to several pathologies, this study proposes a method to assess if mutational burden could be the cause of the pathological phenotype in patients who are negative for Mendelian genetic testing and who carry multiple recessive variants, thereby identifying candidate oligogenic gene combinations.^[24,25]^ The preliminary mathematical model integrates bioinformatics and computational biology techniques to quantify the mutational burden in IRDs. The construction of this model required compiling a comprehensive list of known retinal dystrophy-associated genes and a list of biological pathways enriched by these genes. For model development, two semi-synthetic datasets were used, each reporting clinical and genetic data of patients affected by retinal dystrophies (cases) or patients affected by other genetic diseases (controls). The model assessed the cumulative impact of genetic variants within the selected genes and the identified molecular pathways.

Key findings include significant differences in “the canonical retinoid cycle in rods (twilight vision)” (*P* = 0.012084) and “retinoid cycle disease events” (*P* = 0.00002). These pathways exhibited a highly significant difference compared to other pathways, suggesting a higher pathway activity in the case group compared to the control group. Both pathways were found to be significantly more activated in the case group than in the control group, according to the Mann–Whitney U test, and showed a high correlation (*r* = 0.92). The KDE model was fitted to the standardized feature values, and we calculated the percentile ranks for each patient's standardized feature value. Finally, we established a 90 ${}^{\text{th}}$ -percentile threshold to classify patients based on their mutational burden.

The correlation of these variants and their interpretation, which cannot always adhere to the Mendelian model, remains one of the challenges of Next-Generation Sequencing (NGS), and it has paved the way for research into new models of interaction and inheritance.^[36,37]^ The rationale that variants in different genes that are part of the same molecular network can influence phenotypes is becoming increasingly evident from system biology studies. These investigations reveal the presence of a specific genetic architecture that, if disturbed, can lead to pathological phenotypes, including retinal disorders.^[38,39]^ Apart from identifying the association between mutational burden and retinal conditions, our developed mathematical model uncovers gene combinations that remain hidden under the classic Mendelian paradigm, yet appear in cases but not in controls. These combinations could identify genes with oligogenic effects, thereby uncovering new etiological patterns. Similarly, a study by Pounraja et al developed a general framework for identifying oligogenic combinations in complex disorders.^[37]^

Our model identified two combinations (*ABCA4*, *RDH12*) and (*ABCA4*, *RBP3*) present in cases and not in controls. Although the difference is not statistically significant, the participation of *ABCA4* suggests a possible oligogenic effect—considering that *ABCA4* has already been proven to have an epistatic and oligogenic effect with *ROM1*, *PROM1,* and *PRPH2*.^[39,40,41]^ Despite advances in NGS and data science, identifying meaningful combinations remains challenging; the effect of individual variants may be both summative and subtractive, and such studies require large case series.^[42,43,44]^ In addition, functional and independent studies should validate the results obtained.^[42,43]^

Our proposed model has some limitations that warrant consideration. First, the model relies heavily on existing protein–protein interaction networks and genetic databases, which may contain inherent biases and inaccuracies. Second, the quality and completeness of genetic variant data vary across different populations, which may impact the performance of mutational burden analysis when applied to other datasets. Third, population-specific variability in variant frequencies across ethnic groups may introduce systematic biases in the estimation. Implementing standardized variant-curation procedures and integrating population-stratified reference datasets may improve the robustness of the model. Fourth, the complexity of retinal dystrophies, including their phenotypic heterogeneity and variable expressivity, poses challenges to accurate diagnosis and gene discovery. Finally, the weights of pathogenicity levels were arbitrarily determined; nevertheless, given the preliminary nature of our study, the methodological approach is more critical than the specific weighting system.

Future studies should explore alternative weighting schemes and evaluate these across different cohorts to validate and refine the robustness of our results. The generalizability of our model to other genetic disorders and clinical settings remains to be evaluated, highlighting the need for further refinement and validation in diverse patient cohorts. Our study is in line with other published articles that show the use of machine learning and network analysis can help investigate the genetic bases of inherited diseases, advancing beyond the classical Mendelian inheritance model.^[24]^ If validated through further studies, the mutational burden calculation and the identification of unique gene combinations could serve as a critical starting point for exploring potential oligogenic inheritance patterns. Such an approach has already proven valuable in several diseases, offering an analogous rationale for explaining complex phenotypes through the interplay of multiple variants rather than a single causative mutation.^[43,44,45,46]^

In summary, retinal dystrophies represent a heterogeneous group of genetic disorders that collectively lead to progressive degeneration of the retina, resulting in vision loss and impairment. Despite the identification of numerous disease-causing genes, a substantial proportion of patients with retinal dystrophies receive negative results from genetic testing. This study shows that mutational load in affected subjects is statistically associated with the pathological condition in some patients; therefore, our proposed algorithm can be used to analyze new subjects and classify them as positive or negative for burden effect. Moreover, we identified combinations that were unique to the case group and which allowed us to propose new combinations that need to be validated in further studies.

##  Financial Support and Sponsorship

None.

##  Conflicts of Interest

All affiliations of the authors with private companies have been declared to make clear the position regarding the interests of these companies. The authors are affiliated with private companies, which could pose a conflict of interest. The authors of this article are reported to be patent inventors.
